# Vitamin B12 status and the risk of developing sepsis in patients with bacterial infection: a prospective observational cohort study

**DOI:** 10.1186/s12916-024-03552-3

**Published:** 2024-08-13

**Authors:** Andreas Pregernig, Ulrike Held, Martin Schläpfer, Beatrice Beck-Schimmer

**Affiliations:** 1 Institute of Anesthesiology, University Hospital Zurich, Rämistrasse 100, Zurich, CH-8091 Switzerland; 2 Epidemiology, Biostatistics and Prevention Institute, University of Zurich, Hirschengraben 84, Zurich, CH-8001 Switzerland

**Keywords:** Sepsis, Infection, Vitamin B12, Holotranscobalamin, Methylmalonic acid, Prospective, Observational study

## Abstract

**Background:**

Data have shown that vitamin B12 has immunomodulatory effects via different pathways, which could influence the pathophysiology of sepsis. The objective of this study was to investigate whether vitamin B12 levels, assessed by the measurement of holotranscobalamin (HTC), total vitamin B12 (B12), and methylmalonic acid (MMA, which accumulates in case of B12 deficiency), are associated with the development of sepsis in patients with onset of bacterial infection.

**Methods:**

This was a single-center, prospective observational pilot study. Adult patients who presented to the emergency department with bacterial infection confirmed by a positive microbiological culture result were included in the study and followed up for 6 days to assess whether they developed sepsis or not. The primary objective was to compare HTC concentration in patients who developed sepsis to those who did not develop sepsis. Secondary objectives were the evaluation of B12 and MMA concentrations in those two groups. Multiple logistic regression models were used, with presence of sepsis as the outcome variable, and HTC, B12, and MMA concentrations as predictor variables, separately, and adjusted for potential confounders.

**Results:**

From 2019 to 2022, 2131 patients were assessed for eligibility, of whom 100 met the inclusion criteria. One patient was excluded from the analysis due to missing data. Of the 99 patients, 29 developed sepsis. There was no evidence for an association between HTC or B12 concentration and the development of sepsis (OR 0.65, 95% CI 0.31–1.29, *p* = 0.232, OR 0.84, 95% CI 0.44–1.54, *p* = 0.584, respectively). There was an association between MMA concentration and the development of sepsis, with a positive effect, i.e. with increasing MMA, the odds for sepsis increased (OR 2.36, 95% CI 1.21–4.87, *p* = 0.014). This association remained significant when adjusted for confounders (OR 2.72, 95% CI 1.23–6.60, *p* = 0.018).

**Conclusions:**

Our study found an association between elevated MMA concentration and the development of sepsis. We did not find an association between HTC and B12 concentrations and the development of sepsis. Further, larger studies are warranted, as it could lead to interventional trials investigating whether B12 supplementation provides a clinical benefit to patients with infection or sepsis.

**Trial registration:**

The study was registered on ClinicalTrials.gov under the identifier NCT04008446 on June 17, 2019.

**Supplementary Information:**

The online version contains supplementary material available at 10.1186/s12916-024-03552-3.

## Background

Sepsis affects millions of people throughout the world [1]. To this day, it is still one of the main causes of death in intensive care units (ICUs), with high mortality rates across all countries [2, 3]. Sepsis pathophysiology is characterized in part by excessive generation of potent proinflammatory cytokines and chemokines, reactive oxygen species, and nitric oxide (NO), as well as redox imbalance and widespread immunothrombosis which can result in disseminated intravascular coagulation [4, 5].

Malnutrition is well known to interfere with the immune system and constitute a risk factor for infection [6, 7]. But there is also increasing evidence that deficiency or marginal deficiency in micronutrients (vitamins and trace elements) is linked with an altered immune function and susceptibility to infection or sepsis [8–10].

Vitamin B12 (cobalamin) is a water-soluble vitamin that is obtained from animal dietary sources. Three quarters of vitamin B12 circulate in the blood bound to haptocorrin, and one quarter is bound to transcobalamin to form holotranscobalamin (HTC), the physiologically active form of B12 presented for cellular uptake [11].

In the cell, B12 is required by two enzymes [12]: methylmanolyl-CoA mutase which converts methlymalonic acid (MMA) to succinyl-CoA, an intermediary in the Krebs cycle, and methionine synthase which converts homocysteine to methionine, which is essential for protein synthesis and is involved in cellular methylation reactions (e.g., methylation of DNA, histones and other regulators of gene expression). Total vitamin B12, HTC, as well as MMA and homocysteine, which accumulate in case of B12 deficiency, can be measured in the blood to determine vitamin B12 status, although homocysteine has low specificity for this use as other causes can lead to its accumulation [11].

Vitamin B12 deficiency is frequent and occurs in 6% of people under 60 years, and 20% in people over 60 [13, 14]. Marginal or subclinical deficiency is even more common, but its clinical relevance is still unknown [15–17]. While vitamin B12 deficiency is mainly known for its hematological and neurological manifestations, some data have suggested a link between vitamin B12 and inflammation. Cobalamin has notably been shown to act as an antioxidant, suppress cytokine production, and regulate NO synthase activity [18–20]. A study also demonstrated that vitamin B12 deficiency increases tumor necrosis factor-alpha and lowers epidermal growth factor in cerebrospinal fluid and serum [21]. In patients suffering from sepsis, methionine metabolites and homocysteine (both linked to vitamin B12) have been shown to be abnormal [22, 23]. An animal model further indicated that low vitamin B12 levels are correlated with susceptibility to an infectious challenge [24]. Data regarding human subjects are still scarce.

Vitamin B12 deficiency is relevant today and may increase in the near future due to vegetarianism, veganism, the use of proton pump inhibitors or metformin, gastro-intestinal surgery, and an overall aging population [13, 25, 26]. An association between low vitamin B12 status and the occurrence of sepsis could lead to interventions aiming to increase vitamin B12 levels thus lowering the risk of sepsis in patients with infection.

In this study, we assessed if vitamin B12 levels, determined by the measurement of HTC, vitamin B12, and MMA, were associated with the development of sepsis, in patients admitted to the emergency department (ED) with onset of microbiologically confirmed bacterial infection. The hypothesis is that patients with positive cultures developing sepsis have lower vitamin B12 levels than those who do not develop sepsis.

## Methods

This was a single-center prospective observational cohort study which took place at the University Hospital Zurich (Zurich, Switzerland). It was approved by the local ethics committee (KEK ZH 2019 number 00702) and was registered on ClinicalTrials.gov under the identifier NCT04008446 on June 17, 2019.

### Study participants

Adult patients (≥ 18 years old) presenting to the ED with fever (≥ 38 °C) and a quick Sequential Organ Failure Assessment score (qSOFA) of ≤ 1 who had a positive microbiological culture result were included in the study.

Pregnant or breastfeeding patients, and patients unable to provide informed consent were excluded from the study.

### Study design

As there are few data about vitamin B12 status in patients with sepsis, this was a pilot study. On weekdays (Monday to Friday), consecutive patients who were admitted to the ED with suspicion of infection were screened, and those with fever (≥ 38 °C), qSOFA ≤ 1, and for whom microbiological cultures were ordered or sampled were prospectively enrolled. A member of the study team asked the patients for their written consent for sampling of one blood vial and participation in the study. If consent was obtained, one blood sample was obtained in the ED. This blood sample was centrifuged, and the serum aliquot was stored pending the results of the microbiological cultures. If at least one of the culture results was positive for bacterial infection, the patient was included in the study and the serum was analyzed. If all cultures were negative, the serum was discarded and the patient was not included in the study. Patients were followed for 6 consecutive days after ED admission to assess whether they developed sepsis or not. After 6 days, they were either stratified as bacterial infection with sepsis or bacterial infection without sepsis. Sepsis was defined according to the Sepsis-3 definitions [27].

### Determination of HTC, B12, and MMA plasma concentrations

One blood sample (red BD Vacutainer® Clot Activator Tube) was obtained in the ED by a member of the study team. As soon as possible after allowing the blood to clot (20 to 30 min), the blood sample was centrifuged and the serum aliquot was stored in a freezer at a temperature of − 20 °C and protected from light exposure. For patients who had a positive microbiological culture, serum concentrations of HTC, B12, and MMA were then determined by the Institute of Clinical Chemistry of the University Hospital Zurich. The assays used for the measurements were Active-B12 (with Architect i200SR device, by Abbott) for HTC, Elecsys Vitamin B12 II (with Cobas 8000 e801 Module, by Roche) for B12, and MassChrom® Methylmalonic Acid (by Chromsystems, with 4500MD mass spectrometer by Sciex) for MMA. All the assays used are commercial and successfully underwent external quality control and proficiency testing.

### Objectives and outcomes

The primary objective of our study was to compare the HTC concentration in patients who developed sepsis to those who did not develop sepsis. Secondary objectives were the evaluation of vitamin B12 and MMA concentrations in those two groups.

### Statistical analyses

The sample size was calculated to detect a difference in HTC concentration with a predefined precision. A difference in HTC concentration with a 95% confidence interval width of 20 pmol/l yielded a sample size of 100 patients.

For the results, multiple logistic regressions models were used, with presence of sepsis as the binary outcome variable, and independent variables HTC, vitamin B12, and MMA concentrations, separately, and adjusted for potential confounders selected on the basis of clinical knowledge (malnutrition, defined as medium or high risk of malnutrition according to MUST score [28], age, diabetes). Continuous data were presented as mean and standard deviation or median and interquartile range (IQR) if not normally distributed. Categorical data were presented as numbers (*n*) and proportions (%) of total. Results were presented as odds ratios with 95% confidence intervals. In addition, thresholds to define B12 deficiency vary between different countries and centers [14], and while the study objective was to compare HTC, B12, and MMA concentrations regardless of any threshold, we compared the proportion of patients with B12 deficiency and marginal deficiency according to the latest published NICE guidelines [29]. The statistical programming language R, version 4.2.2, was used for all statistical analyses [30]. The study was performed in accordance with the Strengthening the Reporting of Observational studies in Epidemiology (STROBE) guidelines (Additional file 1) [31].

## Results

**Fig. 1:**
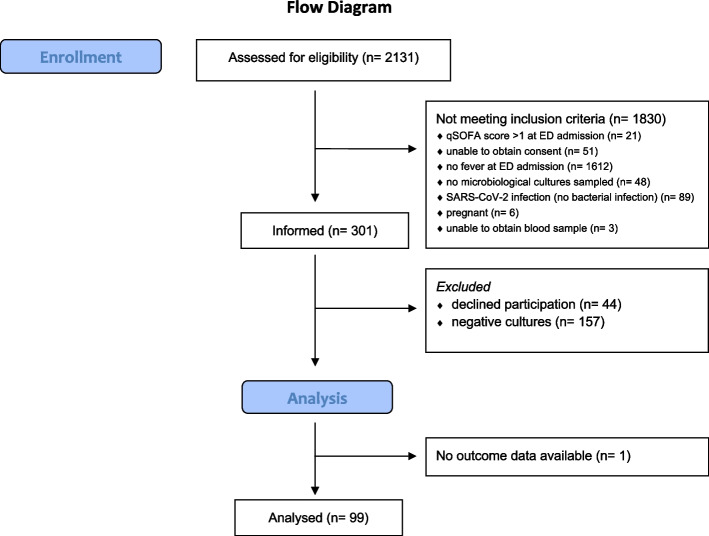
STROBE flow diagram

From June 2019 to November 2022, 2131 patients were assessed for eligibility. 1830 patients did not meet the inclusion criteria. Of the remaining 301 patients, 44 declined participation in the study, 157 were excluded due to negative microbiological culture results, and 100 were included in the study. One patient was excluded from the analysis due to missing data (transfer to another hospital) that precluded formal adjudication of whether he developed sepsis or not. The other 99 patients were all included in the statistical analysis. A flow diagram of the study is shown in Fig. 1.

The patients’ baseline characteristics and relevant comorbidities are shown in Table 1. The mean patient age was 59 years. Only 4 patients were receiving vitamin B12 supplementation prior to ED admission. 20 patients were deemed at high risk of malnutrition, with a Malnutrition Universal Screening tool (MUST) score of 2 points or more. The majority of patients had a genito-urinary source of infection, and the second most frequent source of infection was intra-abdominal. Of the 99 patients included in the study, 29 developed sepsis. The proportion of patients who had a qSOFA score of 1 at baseline was higher in the group of patients who developed sepsis, but baseline characteristics between patients who developed sepsis and those who did not develop sepsis were balanced, with no other statistically significant difference.

**Table 1:** Patients baseline characteristics

	All patients (*n* = 99)	No-sepsis (*n* = 70)	Sepsis (*n* = 29)	SMD	p*
Age (mean (SD))	58.60 (18.56)	57.26 (18.54)	61.83 (18.51)	0.247	0.267
Female (%)	46 (46.5)	36 (51.4)	10 (34.5)	0.347	0.188
Male (%)	53 (53.5)	34 (48.6)	19 (65.5)	0.347	0.188
Ethnicity (%)				0.327	0.640
Asian	3 (3.0)	2 (2.9)	1 (3.4)		
Black	3 (3.0)	3 (4.3)	0 (0.0)		
Hispanic	2 (2.0)	1 (1.4)	1 (3.4)		
White	91 (91.9)	64 (91.4)	27 (93.1)		
Received antibiotics before blood cultures sampled (%)	26 (26.3)	16 (22.9)	10 (34.5)	0.259	0.344
Obesity (%)				0.369	0.320
No	77 (77.8)	52 (74.3)	25 (86.2)		
Yes	12 (12.1)	9 (12.9)	3 (10.3)		
NA	10 (10.1)	9 (12.9)	1 (3.4)		
Smoking (%)				0.456	0.076
No	89 (89.9)	66 (94.3)	23 (79.3)		
Yes	7 (7.1)	3 (4.3)	4 (13.8)		
NA	3 (3.0)	1 (1.4)	2 (6.9)		
Past transplantation (%)	20 (20.2)	15 (21.4)	5 (17.2)	0.106	0.844
Chronic kidney disease (%)	17 (17.2)	12 (17.1)	5 (17.2)	0.003	1
Diabetes (%)	19 (19.2)	15 (21.4)	4 (13.8)	0.201	0.550
Cancer (%)	32 (32.3)	23 (32.9)	9 (31.0)	0.039	1
Liver disease (%)	13 (13.1)	6 (8.6)	7 (24.1)	0.431	0.078
Pancreatic disease (%)	2 (2.0)	2 (2.9)	0 (0.0)	0.243	0.893
Medication					
Vitamin B12 supplementation (%)	4 (4.0)	2 (2.9)	2 (6.9)	0.188	0.713
PPI or other antacids (%)	40 (40.4)	26 (37.1)	14 (48.3)	0.227	0.422
Metformin (%)	9 (9.1)	8 (11.4)	1 (3.4)	0.308	0.383
Immune suppressors (%)	34 (34.3)	23 (32.9)	11 (37.9)	0.106	0.802
Risk of malnutrition according to MUST score (%)				0.399	0.360
Low risk (0 pts)	54 (54.5)	42 (60.0)	12 (41.4)		
Medium risk (1 pt)	7 (7.1)	5 (7.1)	2 (6.9)		
High risk (≥ 2 pts)	20 (20.2)	12 (17.1)	8 (27.6)		
NA	18 (18.2)	11 (15.7)	7 (24.1)		
qSOFA score = 1 (%)	34 (34.3)	17 (24.3)	17 (58.6)	0.744	0.002
Infection focus (one patient may have more than one focus)					
Genito-urinary (%)	50 (50.5)	37 (52.9)	13 (44.8)	0.161	0.613
Intra-abdominal (%)	20 (20.2)	13 (18.6)	7 (24.1)	0.136	0.724
Pulmonary (%)	11 (11.1)	6 (8.6)	5 (17.2)	0.261	0.369
Skin/soft tissue/catheter (%)	6 (6.1)	4 (5.7)	2 (6.9)	0.049	1
Other (%)	4 (4.0)	4 (5.7)	0 (0.0)	0.348	0.451
Unknown (%)	11 (11.1)	8 (11.4)	3 (10.3)	0.035	1

*n* number, *SMD* standardized mean difference, *p p*-value*, SD* standard deviation, *NA* missing data, *PPI* proton pump inhibitor, *MUST* Malnutrition Universal Screening Tool, *qSOFA* quick Sequential Organ Failure Assessment score

^*^Exploratory *p*-values

**Fig. 2:**
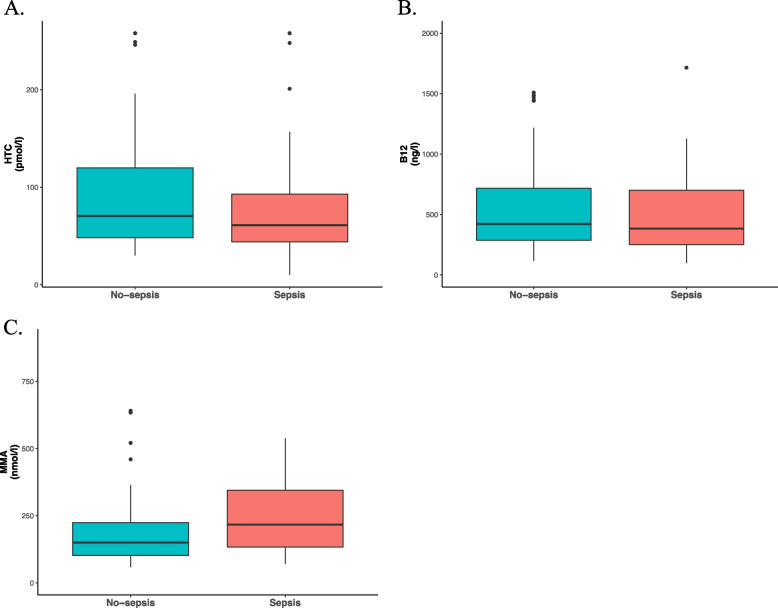
HTC, B12, and MMA concentrations, in patients stratified according to whether or not they developed sepsis

The median HTC concentration in patients who developed sepsis was 61 pmol/l (IQR 44–93), and 71 pmol/l (IQR 48–120) in patients who did not develop sepsis (*p* = 0.319). B12 and MMA concentrations in the no-sepsis group compared to the sepsis group were 421 ng/l (IQR 287–717) and 384 ng/l (IQR 250–701) (*p* = 0.541), and 150 nmol/l (IQR 102–224) and 217 nmol/l (IQR 133–345) (*p* = 0.018), respectively. HTC, B12, and MMA values are depicted in depicted in Fig. 2.

Multiple logistic regression models were fitted with sepsis as the outcome variable and either HTC, B12, or MMA as predictor variables. In the analysis, there was no association between HTC or B12 concentration and sepsis. We did find an association between MMA concentration and the development of sepsis, with a positive effect, i.e., with increasing MMA, the odds for sepsis increased. The models are summarized in Table 2.

**Table 2:**
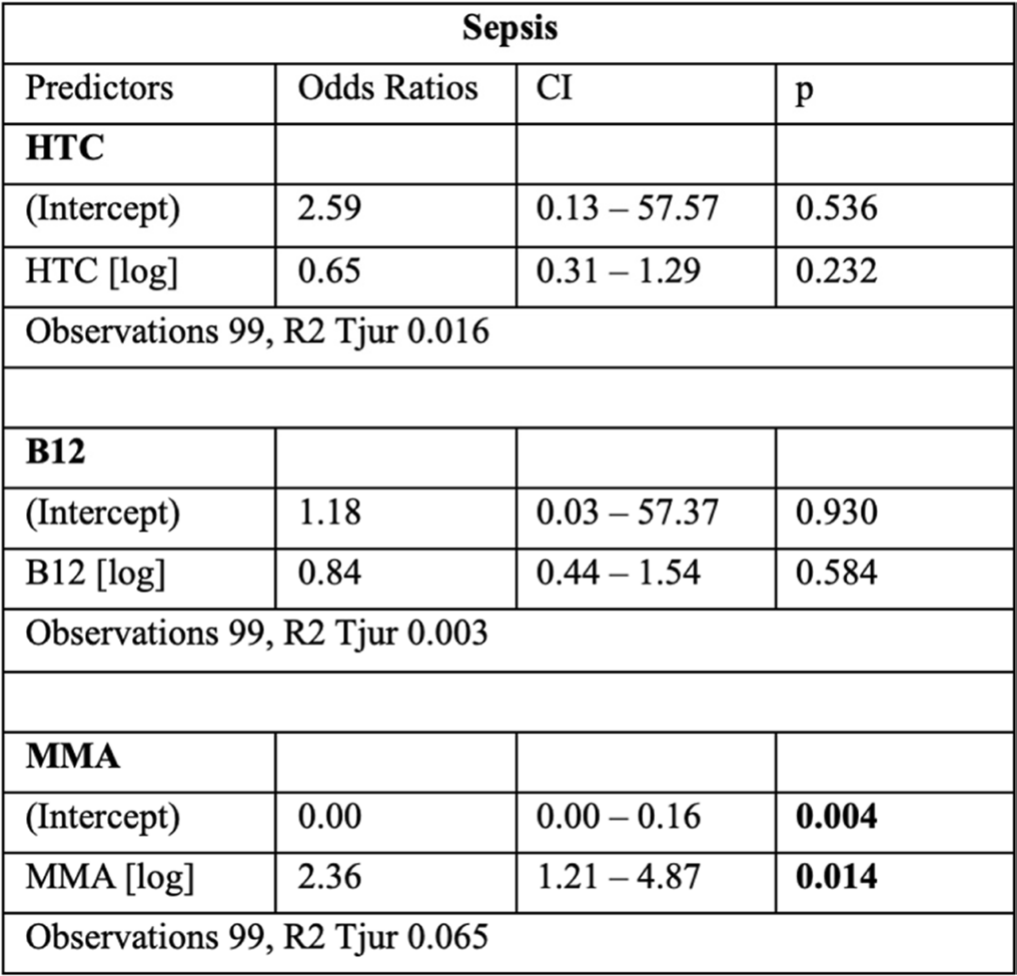
Results of the logistic regression models, unadjusted

*CI* confidence interval, *p* *p*-value

In addition, to control for potential confounding, the variables age, diabetes, and malnutrition were included in the aforementioned models. While there was still no evidence for an association between HTC or B12 and sepsis, the association between MMA and sepsis remained significant when confounders were included in the logistic regression model. These models are presented in Table 3.

**Table 3:**
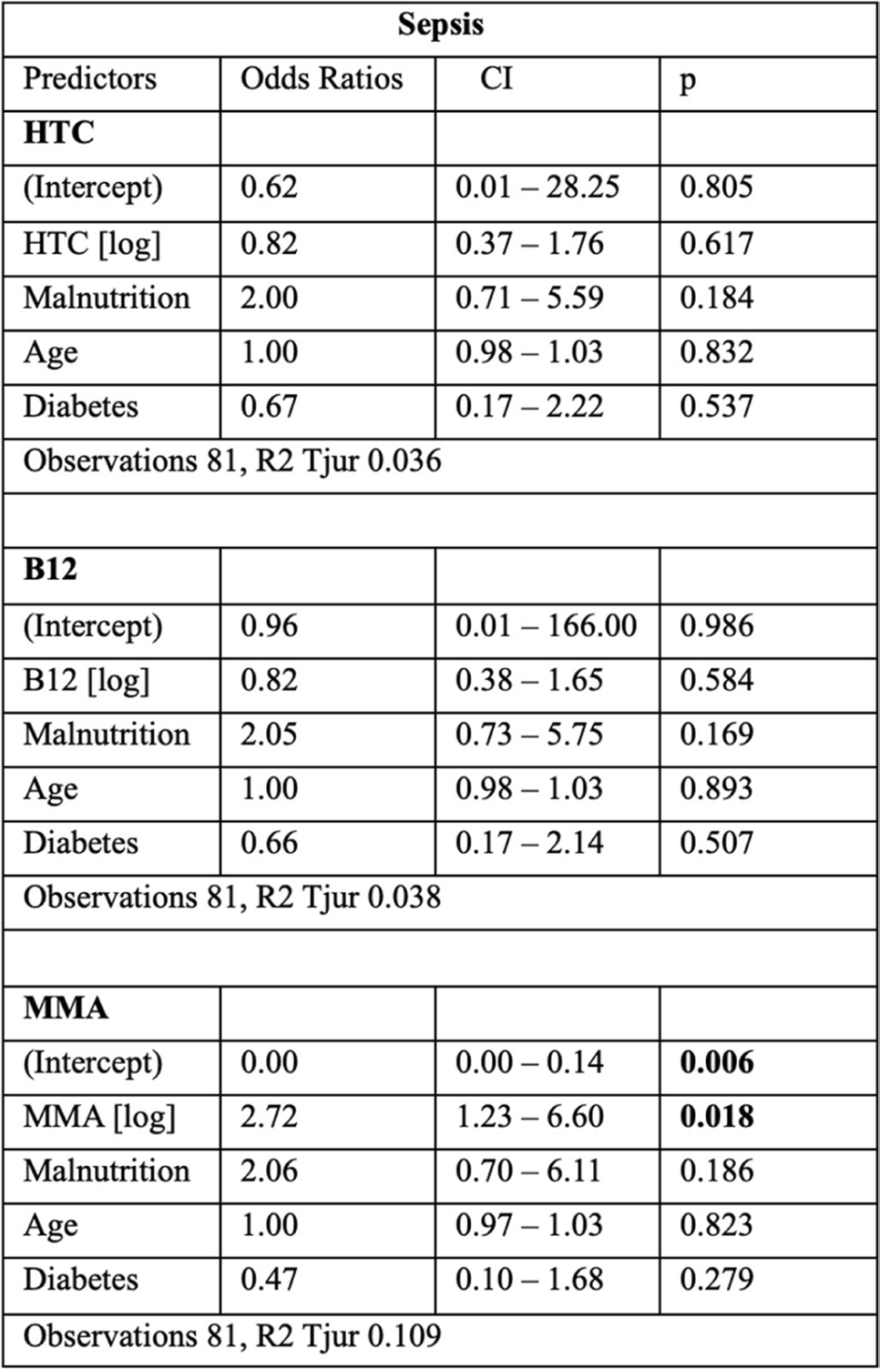
Results of the logistic regression models, adjusted for malnutrition (defined as medium or high risk according to MUST score), age, and diabetes

*CI* confidence interval, *p* *p*-value

Finally, we compared the proportion of patients with B12 deficiency and marginal deficiency according to the latest published NICE guidelines. The proportion of patients with B12 deficiency defined by MMA ≥ 360 nmol/l (in patients 65 years old or more) or MMA ≥ 280 nmol/l (in patients 64 years old or less) was significantly higher in the group of patients with sepsis, compared to those who did not develop sepsis. There was no significant difference when using thresholds of HTC < 25 pmol/l or B12 < 180 ng/l for deficiency, or HTC 25–70 pmol/l and B12 180–350 ng/l for marginal deficiency. The results are detailed in Table 4.

**Table 4:**
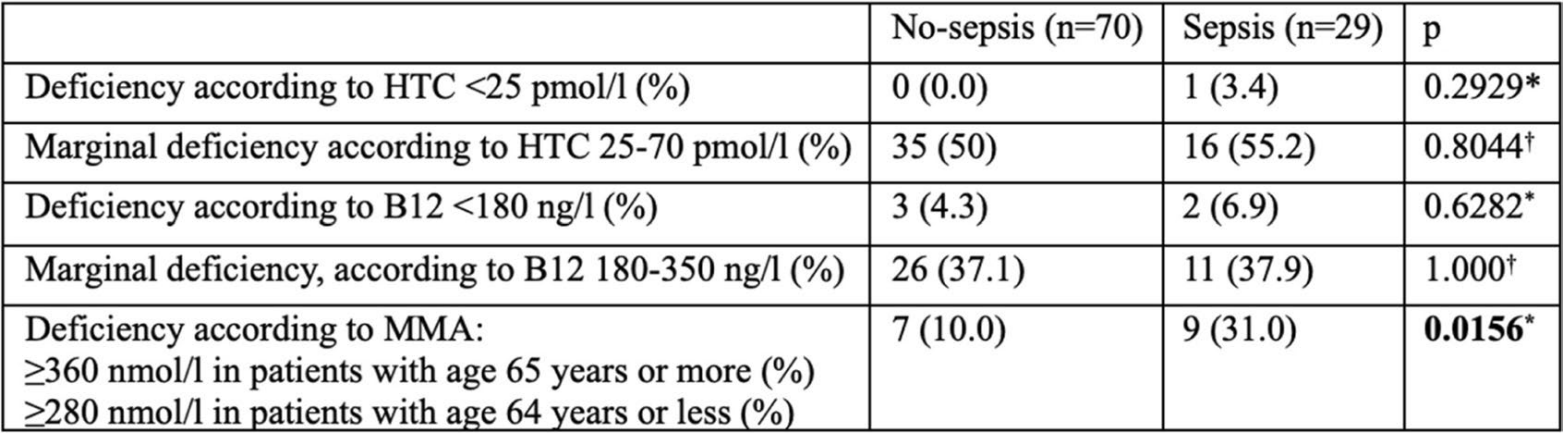
Proportion of patients with B12 deficiency and marginal deficiency according to the thresholds from the 2024 Vitamin B12 deficiency NICE guideline summary

*According to Fisher’s exact test

†According to Pearson’s chi-squared test with Yates’ continuity correction

## Discussion

This study investigated whether vitamin B12 status, assessed by the measurement of HTC, vitamin B12, and MMA, was associated with the development of sepsis in patients who present to the ED with onset of bacterial infection. The results of our study show an association between elevated MMA concentration and the development of sepsis. This association remained significant when potential confounders were included in the analysis. We did not find evidence for an association between HTC or total vitamin B12 concentration and development of sepsis.

The role of micronutrients and vitamins in the pathophysiology of sepsis has been thoroughly researched in recent years. A part of the appeal of these molecules is their generally favorable safety profile when used as a treatment regimen, as well as their availability [32]. Some of the most rigorously researched micronutrients include vitamin C, B1, and vitamin D. Early data supported that low vitamin C was associated with worse outcome in sepsis, and that treatment with vitamin C, used in isolation or in combination with vitamin B1 and other agents, might reduce organ damage and mortality in septic patients [33–35]. The latest studies are more conflicting, and the evidence for a clinical benefit remains unclear [36–40]. Similarly, vitamin D deficiency was associated with increased susceptibility to infections and worse outcome in sepsis patients, but randomized trials assessing vitamin D have shown mixed results [41–44]. Vitamin B12 has been comparatively less investigated than other vitamin micronutrients, with scarce human data available, mostly from retrospective sources, and no randomized trial to this date, despite promising preclinical studies [45, 46]. However, recently, a phase 2 pilot randomized controlled trial evaluated high-dose intravenous hydroxycobalamin as a treatment regimen in septic shock patients, with results showing that the conducting of larger phase-2 and phase-3 trials should be feasible, as well as promising clinical results (lower vasopressor use in the group of patients treated with B12, absence of serious adverse events) despite the small sample size, although these were not the main outcomes of the study [47].

The strengths of our study are its prospective nature, and that we included patients at onset of bacterial infection, when they first presented to the ED, allowing us to take the blood samples as soon as they entered the ED and thus very early in the development of disease, before administration of any fluids or other treatments which could interfere with the measurement of B12 status. For this reason, we excluded patients who were overtly in septic shock (qSOFA > 1) and who might have already received large quantities of fluids in the pre-hospital setting. We also evaluated vitamin B12 status not only through total vitamin B12 concentration but also with HTC and MMA concentrations, which allows a more precise assessment of vitamin B12 status [11].

Our study also has limitations. This being a pilot study, the sample size is quite low. With very few clinical data available, we had to opt for a precision-based sample size calculation rather than a power-based calculation. Focusing on patients admitted to the ED allowed us to include patients at the very beginning of the onset of bacterial infection, but we did not recruit patients who developed infections while hospitalized in medical or surgical units, which could have also provided insightful data. We drew blood samples at admission to the ED before the results of microbiological cultures were known, exclusively in patients who had fever (≥ 38.0 °C). This ensured we only sampled blood in patients who were highly likely to develop positive cultures, thereby preventing the sampling and discarding of large amounts of blood samples which would be unethical and logistically unfeasible due to the high number of patients. These inclusion criteria (presence of fever and microbiologically confirmed bacterial infection) entail that some patients could not be included (such as patients with bacterial infection but who presented to the ED without fever), which might affect generalizability, but they were necessary to limit the heterogeneity of the patient population, given that this was a pilot study with a sample size of 100 patients. We also showed that the proportion of patients with B12 deficiency was higher in the group of patients who developed sepsis when using MMA ≥ 360 nmol/l (in patients 65 years old or more) or MMA ≥ 280 nmol/l (in patients 64 years old or less) as a threshold, but these results should be interpreted with caution as there was no difference when using thresholds based on HTC or B12 concentration for deficiency or for marginal deficiency, and this was not a main outcome for which this study was powered. Our study was performed in Switzerland and most included patients are of white ethnicity. Some geographical regions, including Latin America, Asia, and Africa, have a much higher prevalence of B12 deficiency, sometimes as high as 80% [17], and investigating the association between B12 deficiency and sepsis in such regions might provide different results. Prevalence of B12 deficiency also varies according to ethnicity within a single geographical region, and studies have shown great variability between different ethnic groups within regions such as the USA, UK, and New Zealand [48, 49]. Some authors have even suggested the need for reference intervals adjusted for specific ethnicities [50]. Having a study population composed of over 90% of whites prevented ethnic heterogeneity from significantly affecting the investigation of the association between B12 deficiency and sepsis, albeit at the cost of limited insight into other ethnicities.

Sepsis is incredibly complex with numerous demographic, genetic, and clinical factors influencing its development and pathophysiological pathways [4]. The results of our pilot study are promising and could indicate that vitamin B12 status might have an implication in the development of sepsis. Of the markers of B12 status, only the difference in MMA was statistically significant. While HTC and B12 concentrations were lower in the sepsis group compared to the no-sepsis group, the differences were not significant. Larger prospective studies might provide enough power to detect a difference in absolute B12 and HTC concentrations.

In addition, not only outright deficiency but subclinical deficiency and functional deficiency might be a risk factor for sepsis. Cutoff thresholds in B12 or HTC values to define deficiency vary between countries and hospitals. There is a range between deficiency and normal values, known as marginal deficiency, which is of yet unknown clinical significance, and which could be a risk factor for sepsis [51]. Normal B12 or HTC values also do not exclude deficiency. A technically normal value may, depending on individual variability, be insufficient in a specific patient, as shown by the accumulation of MMA and homocysteine in the blood. MMA has been described as the best marker for assessing B12 status, as it reflects the function of the vitamin and not just its absolute concentration in the blood [15, 52]. The measurement of MMA and evaluation of subclinical deficiency should be considered in the design of further studies investigating the association between B12 deficiency and sepsis.

## Conclusions

In conclusion, we performed a prospective observational pilot study, assessing if vitamin B12 status is associated with the development of sepsis in patients with the onset of bacterial infection. Our study found evidence of an association between elevated MMA concentration and the development of sepsis. We did not find an association between HTC and B12 concentration and the development of sepsis. Our pilot study encourages further, larger studies, precisely investigating if vitamin B12 deficiency is associated with sepsis, including the measurement of MMA and the evaluation of subclinical deficiency, as well as the derivation of thresholds which could be used in clinical practice. If additional studies also find evidence of this association, it could lead to interventional trials investigating whether B12 supplementation provides a clinical benefit to patients with infection or sepsis.

### Supplementary Information

Additional file 1. STROBE Statement—Checklist of items that should be included in reports of cohort studies.

## Data Availability

The datasets used and/or analyzed during the current study are available from the corresponding author on reasonable request.

## Abbreviations

95% CI: 95% Confidence interval
B12: Total vitamin B12
ED: Emergency department
HTC: Holotranscobalamin
ICU: Intensive care unit
IQR: Interquartile range
MMA: Methlymalonic acid
MUST: Malnutrition Universal Screening tool
NO: Nitric oxide
OR: Odds ratio
qSOFA: Quick Sequential Organ Failure Assessment score
SOFA: Sequential Organ Failure Assessment score
STROBE: Strengthening the Reporting of Observational studies in Epidemiolog
